# Changes in Functional Connectivity of Specific Cerebral Regions in Patients with Toothache: A Resting-State Functional Magnetic Resonance Imaging Study

**DOI:** 10.1155/2020/6683161

**Published:** 2020-12-28

**Authors:** Shi-Nan Wu, Meng-Yao Zhang, Hui-Ye Shu, Rong-Bin Liang, Qian-Ming Ge, Yi-Cong Pan, Li-Juan Zhang, Qiu-Yu Li, Yi Shao

**Affiliations:** Department of Ophthalmology and Stomatology, The First Affiliated Hospital of Nanchang University, Nanchang, 330006 Jiangxi Province, China

## Abstract

**Objective:**

In order to further study the changes of cerebral functional connectivity in patients with toothache (TA), this study used the resting-state functional magnetic resonance imaging (rs-fMRI) technique and degree centrality (DC) analysis method.

**Methods:**

Eighteen TA patients (8 males, 10 females) and 18 healthy individuals of similar age, sex, and educational levels were recruited as healthy controls (HCs) to take part in the study, and all underwent rs-fMRI examination. And DC technology was used to compare the state of their cerebral spontaneous functional activity. In order to compare the average DC values of the TA group and HC group, we used independent two-sample *t*-test and receiver operating characteristic (ROC) curve to compare the difference of DC values between the two groups, so as to distinguish the accuracy of TA diagnosis. Finally, we also carry out Pearson's linear regression analysis.

**Results:**

The TA group showed higher DC values in the right lingual gyrus (RLG), right precentral gyrus, and left middle temporal gyrus (LMTG) than HCs. Moreover, ROC curve analysis indicated that the area under the curve (AUC) of each cerebral region studied had high accuracy. In addition, linear analysis indicated that the DC values of the RLG were positively correlated with the Hospital Anxiety and Depression Scale (HADS) (*r* = 0.844, *p* < 0.001), and the DC values of the LMTG were positively correlated with the visual analogue scale (VAS) (*r* = 0.723, *p* < 0.001).

**Conclusion:**

TA generates abnormal changes in the intrinsic activity patterns of pain-related and vision-related areas of the cerebral cortex, which will be beneficial to reveal the underlying neuropathic mechanisms.

## 1. Introduction

**Figure 1:**
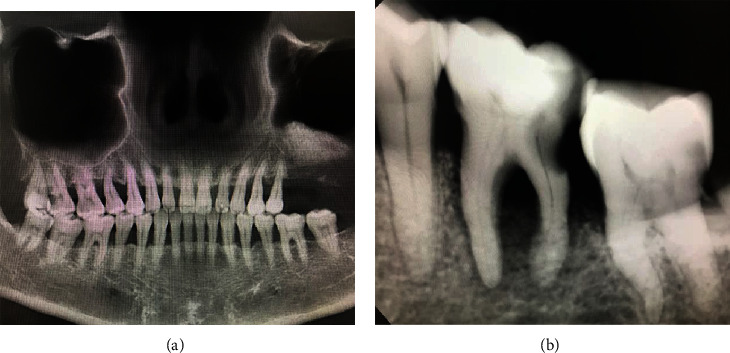
Imaging images of typical toothache. (a) Shows the gross teeth of patients with toothache, while (b) shows the local teeth of patients with toothache.

Toothache (TA) is also called odontalgia, and its pathological mechanism may be odontogenic or nonodontogenic. The odontogenic mechanism includes pain caused by damage to dental pulp and/or periodontal tissues [1] (Figure 1). Nonodontogenic pain includes classic trigeminal pain, posttraumatic trigeminal pain, and persistent idiopathic facial pain. And some specific ectopic lesions cause toothache including pain caused by central lesions, often involving specific sensory nerve pathway damage. Since abnormal cerebral functions can lead to pain, there are few studies on whether TA can reverse the corresponding changes in cerebral cortex function. Pain is a conscious experience of hurtful stimuli and is influenced by memory, emotional, and cognitive factors [2]. Severe TA can be a painful condition that affects sleep, eating, and other daily activities. At present, there are a number of scholars who continue to study the changes of oral neuroscience and corresponding cerebral functional activities through neuroimaging techniques and further reveal the underlying neuropathological mechanism.

In recent decades, with the rapid advancement of imaging technology, resting-state functional magnetic resonance imaging (rs-fMRI) has been widely used in the study of diseases related to cerebral functional activities due to its high spatial resolution and noninvasive advantages. The technique allows researchers to observe changes in neural activity in specific cerebral regions [3, 4]. And as functional magnetic resonance imaging continues to evolve, researchers have gradually uncovered changes in cerebral activity in pain-related disorders [5, 6]. Patients with trigeminal neuralgia exhibited blood-oxygen-level-dependent (BOLD) activation in specific cerebral regions such as the somatosensory cortex [7]. A similar BOLD signal increase was discovered in the primary somatosensory cortex during dental electrical stimulation [8]. In a study of changes in cerebral functional activity in patients with low back pain, functional connectivity (FC) was reduced in the precuneus and significantly enhanced in the medial prefrontal cortex and anterior cingulate gyrus [9]. In another study of patients with acute eye pain, FC in the left superior frontal gyrus and precuneus was also significantly increased [10].

Voxel-wise degree centrality (DC) is one of the important methods widely used to evaluate changes in cerebral network functional activity [11]. The DC method can detect the functional importance of different nodes in the brain at the voxel level [12]. And the DC values represent the degree of direct functional connection between the node and other nodes. As a result, the technique has been applied to study the underlying pathophysiological mechanism of the link between cervical dystonia, schizophrenia, and other diseases and the brain [13, 14]. Meanwhile, the DC method has also been used in the study of many pain-related diseases [15–19] (Table 1). Therefore, DC is a reliable fMRI technology that has not been applied to TA yet. Our team will use the DC approach to compare cerebral FC between TA patients and healthy controls (HCs).

## 2. Materials and Methods

### 2.1. Subjects

Patients with TA, including 8 males and ten females, were recruited as study participants from China. Inclusion criteria of TA patients were (1) pain in the pulp or periodontal tissues of teeth of either dental origin or nondental origin; (2) acute and chronic toothache; (3) no other pain diseases; (4) capable of MRI examination; (5) no abnormal signal changes were found in the brain during conventional MRI examination; and (6) toothache that cannot be attributed to any other disease but does not have an obvious pathologic cause. Exclusion criteria were (1) headache, temporomandibular disorders, fibromyalgia, back pain, etc.; (2) participants in the first generation that had a family history of pain such as headaches; (3) other brain or psychiatric diseases; and (4) contraindications to MRI.

Eighteen HCs matched for sex, age, and education status also participated in the study, including 8 males and 10 females. Inclusion criteria of HCs were (1) no symptoms of TA; (2) no abnormalities in MRI examination of cerebral parenchyma; (3) no cardiovascular disease, mental illness, or cerebral infarction; (4) no drugs or alcohol addiction; and (5) no contraindications for MRI examination, such as implanted metal devices or a cardiac pacemaker.

All the research processes involved in this study followed the Helsinki Declaration. The Medical Ethics Committee also approved the study. The approval number is cdyfy2017021. In addition, the subjects signed informed consent after they were fully aware of the purpose, content, and potential risks of the study.

### 2.2. Pain Scores

The visual analogue scale (VAS) was applied to the measurement of pain in TA patients. Using a ten-centimeter ruler, the patients rated the degree of pain they experienced on a scale of 0 to 10. The higher the patient's score, the more intense the pain he is currently experiencing [20].

### 2.3. MRI Data Acquisition

All the individuals in the study underwent conventional MRI before the BOLD cerebral function examination of rs-fMRI, and no abnormal signal changes in cerebral structure were observed. The MRI scanning was performed using the 3 T magnetic resonance scanner of an 8-channel phased-array head coil (Trio, Siemens, Munich, Germany). During the entire scanning process, all participants were required to remain awake, while breathing smoothly with their eyes closed. The rs-fMRI scan data was collected by using gradient echo-echo planar imaging sequence. Relevant detailed parameters about the apparatus are as follows: 30 axial slices and 240 functional images (gap: 1 mm; repetition time (TR): 2000 ms; echo time (TE): 40 ms; flip angle: 90°; field of view (FOV): 240 × 240 mm; thickness: 4.0 mm; in-plane resolution: 64 × 64). On top of that, we also obtained cerebral structural images for every individual by using a T1-weighted 3D MP-RAGE sequence (176 structural images; acquisition matrix: 256 × 256; TR: 1900 ms; TE: 2.26 ms; flip angle: 9°; FOV: 240 × 240 mm; thickness: 1.0 mm). The entire scanning process lasted 15 min.

### 2.4. fMRI Data Analysis

Firstly, we used the MRIcro software (http://www.MRIcro.com) to analyze the acquired information. The Data Processing Assistant for rs-fMRI software (http://rfMRI.org/, DPARSF) and the Statistical Parametric Mapping 8 (SPM8) were used to preprocess the acquired data. We rejected the data of the first 10 time points to eliminate any interference of an unsteady magnetic field. The remaining 230 volumes were collected. Subjects with more than 2° of angular displacement and an offset > 2 mm in three dimensions during the whole scanning process were excluded from further consideration. A previous study showed fMRI data analysis in more detail [21].

### 2.5. Degree Centrality

Use REST (http://www.restfmri.net) software for DC analysis. The processing procedure included filtering the preprocessed image (0.01 Hz < *f* < 0.08 Hz) to eliminate all kinds of noise that affected data detection, and then, the DC values of 18 patients with the TA and HC group were calculated. To improve normality, we used Fisher transformation to convert the correlation coefficient into a *Z* value:

(1) $$\begin{array}{c} Z_{i}=\frac{{\text{DC}}_{i}-{\text{mean}}_{\text{all}}}{{\text{std}}_{\text{all}}} \end{array}$$

Among them, *Z*_*i*_ refers to the *Z* score of the *i*^th^ voxel; DC_*i*_ refers to the DC value of the *i*^th^ voxel; mean_all_ refers to the average of all voxels in the cerebral structure; and std_all_ refers to the standard deviation.

### 2.6. Brain-Behavior Correlation Analysis

We have used REST software to divide the specific cerebral regions with different DC values between the two groups into regions of interest (ROI), and then, each original DC value of these regions was averaged over all voxels to calculate the mean DC values of each ROI [22]. And the two groups were compared. What is more, the participants completed the Hospital Anxiety and Depression Scale (HADS) to obtain statistical results. In addition, we collected clinical features such as disease duration and VAS score of TA patients. Therefore, we used GraphPad Prism 8 software to analyze the linear correlation between the DC values of the right lingual gyrus (RLG) and HADS, where *p* < 0.05 indicates significant difference. Meanwhile, we also used the same software for linear analysis of the DC values of the left middle temporal gyrus (LMTG) and VAS score above.

### 2.7. Statistical Analysis

In terms of demography and clinical measurement, SPSS20.0 software (USA, Chicago, Illinois, SPSS) was used to compare the differences between the TA and HC group, and the significance level was set at *p* < 0.05. To study the centrality of functional networks represented by the DC values of different cerebral regions, we adopted the statistical method of independent sample *t*-test. Then, independent two sample *t*-test was performed on the standardized DC values of the TA group and HC group by using REST software. After being corrected by Gaussian random field theory, *p* < 0.05 was set as the threshold for statistical significance. We have confirmed cerebral regions with different DC values between the 2 groups and speculated that differences in DC values can distinguish the TA group from HC group. Therefore, in order to differentiate TA patients from control participants, we analyzed the differential diagnostic value of mean DC values in specific cerebral regions by using receiver operating characteristic (ROC) curve.

## 3. Results

### 3.1. Characteristics of the Study Participants

No significant difference in age was noted between the TA and HC groups (*p* = 0.679). The duration of the TA was 2.04 ± 1.08 years. And Table 2 shows more details of the results.

### 3.2. DC Differences

**Figure 2:**
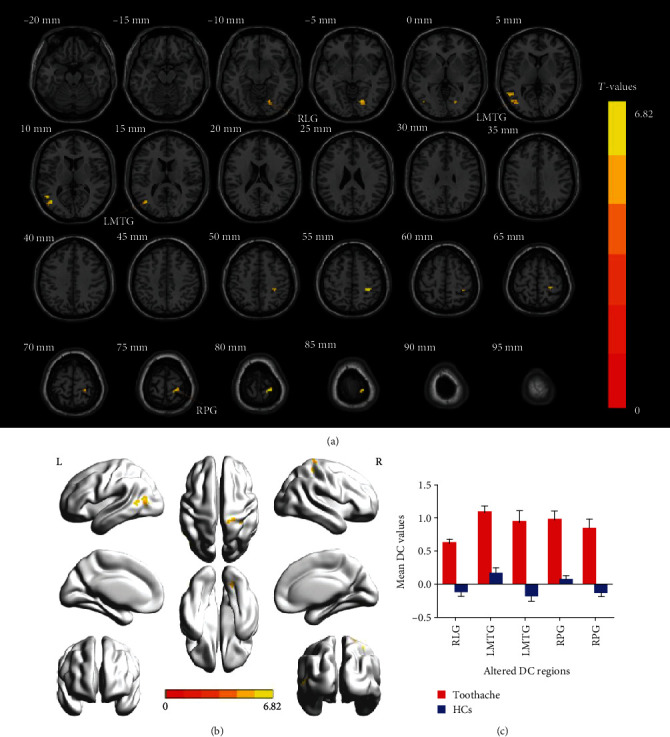
Voxel-wise comparison of DC in the toothache and healthy control group. (a, b) Significant differences in DC were observed. The yellow regions indicate higher DC values. AlphaSim corrected at a cluster size > 40 voxels and a level of *p* < 0.05 for multiple comparisons using Gaussian random field theory. (c) The mean DC values between the TA and HC groups. Abbreviations: DC: degree centrality; TA: toothache; HC: healthy control; RLG: right lingual gyrus; LMTG: left middle temporal gyrus; RPG: right precentral gyrus.

In contrast to HCs, the mean DC values of the RLG (*t* = 6.1635), LMTG (*t* = 6.8246; 6.0745), and right precentral gyrus (RPG) (*t* = 6.5673; 6.6338) in TA patients were significantly higher (Figures 2(a) and 2(b) (yellow) and Table 3). And Figure 2(c) shows the comparison of mean DC values between the TA group and HC group.

### 3.3. ROC Curve

**Figure 3:**
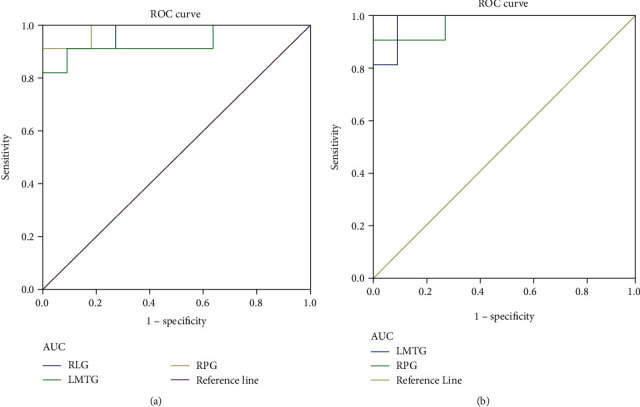
ROC curve analysis of the mean DC values for altered cerebral regions. (a) The area under the ROC curve was 0.975, (*p* < 0.001; 95% CI: 0.918-1.000) for RLG, LMTG 0.934 (*p* = 0.001; 95% CI: 0.818-1.000), and RPG 0.983 (*p* < 0.001; 95% CI: 0.941-1.000). (b) The area under the ROC curve was 0.983 (*p* < 0.001; 95% CI: 0.941-1.000) for LMTG and RPG 0.975 (*p* < 0.001; 95% CI: 0. 918-1.000). Abbreviations: DC: degree centrality; ROC: receiver operating characteristic; RLG: right lingual gyrus; LMTG: left middle temporal gyrus; RPG: right precentral gyrus.

In this study, we hypothesized that differences in changes in DC values in specific cerebral regions between TA patients and healthy subjects could serve as potential markers for differential diagnosis. The results of the final area under the curve (AUC) of DC values in different cerebral regions were as follows: RLG (0.975, *p* < 0.001), LMTG (0.934, *p* = 0.001), and RPG (0.983, *p* < 0.001) (Figure 3(a)); LMTG (0.983, *p* < 0.001) and RPG (0.975, *p* < 0.001) (Figure 3(b)).

### 3.4. Correlation Analysis

**Figure 4:**
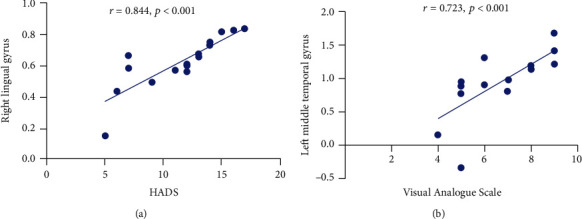
Correlations between the DC values of different regions and the clinical behaviors in the TA group. The DC values in the right lingual gyrus were positively correlated with the HADS (*r* = 0.844, *p* < 0.001); and DC values in the left middle temporal gyrus were positively correlated with the visual analogue scale (*r* = 0.723, *p* < 0.001). Abbreviations: DC: degree centrality; TA: toothache; HADS: Hospital Anxiety and Depression Scale.

The result of the linear analysis showed that the DC values in the RLG were positively correlated with the values of HADS (*r* = 0.844, *p* < 0.001) (Figure 4(a)); and the DC values in the LMTG were positively correlated with VAS (*r* = 0.723,*p* < 0.001) (Figure 4(b)).

## 4. Discussion

**Figure 5:**
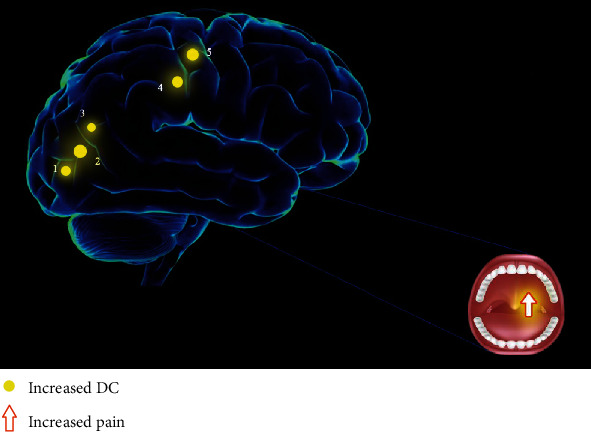
The DC results of cerebral activity in the TA group. Compared with the HCs, the DC values of the following regions were increased to various extents: 1, right lingual gyrus (*t* = 6.1635); 2, left middle temporal gyrus (*t* = 6.8246); 3, left middle temporal gyrus (*t* = 6.0745); 4, right precentral gyrus (*t* = 6.5673); and 5, right precentral gyrus (*t* = 6.6338) in TA patients. The sizes of the spots denote the degree of quantitative changes. Abbreviations: DC: degree centrality; TA: toothache; HC: healthy control.

To our best knowledge, the DC approach is one of the most effective ways to explore changes in cerebral FC. Through our study, by comparing HCs and analyzing the changes of DC values in specific cerebral regions of TA patients, it was found that FC of the RLG, RPG, and LMTG was significantly enhanced (Figure 5).

**Figure 6:**
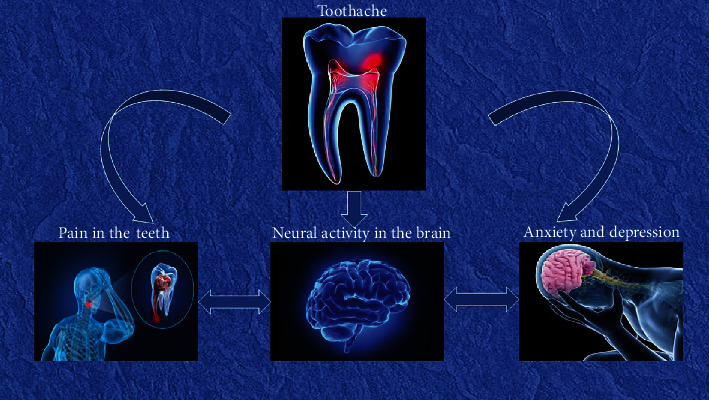
A schematic diagram of the relationship between the effects of toothache on mood and changes in neural activity in related cerebral regions.

The lingual gyrus is one of the cerebral structures involved in logical analysis and the coding of visual memory [23, 24]. In previous studies, Ettlin et al. [8] and Brügger et al. [25] have demonstrated that the lingual gyrus activity significantly increased after dental stimulation. Studies have shown that FC between the lingual gyrus and the dorsal cingulate gyrus was significantly decreased in Alzheimer's patients [26]. Moreover, related studies have also indicated that the lingual gyrus may be an important area for antidepressants and is associated with emotional control [27, 28] (Figure 6). Compared with the normal elderly, the function state of the lingual gyrus and hippocampus affected the thickness of the retinal nerve fiber layer [29]. Another recent FC analysis also revealed an increase in cerebral connectivity in rheumatoid arthritis patients between anterior cingulate cortex and occipital areas [30]. What is more, during tactile stimulation, widespread activations were observed that included the occipital lobe and temporal pole [31]. To support the results discussed above, our experimental results also indicated that the DC values of the RLG were significantly increased in TA patients, indicating that the visual processing function of the RLG was activated, which may be caused by the influence of dental pain. Therefore, this also suggested that changes in lingual gyrus activity in patients with toothache may affect the attention system. Moreover, our linear regression analysis model showed that the DC values of the RLG of TA patients were positively correlated with the HADS values. Through the above analysis, patients with TA may be accompanied by some negative emotions, such as anxiety and depression. The neurological mechanism for this symptom may be the abnormal activation of functional states in this cortical region of the brain.

As a part of the first motor cortex (M1), the precentral gyrus was one of the important structures controlling movement [32, 33]. It has been reported that stronger BOLD responses in the left precentral gyrus, left SI, and right SII were related to the high anxiety during dental pain anticipation [34]. And researches of dental patients displayed increased BOLD signals dominantly in left postcentral gyrus and precentral gyrus [35]. Furthermore, massive studies focused on muscle pain demonstrated that increased activation was observed in M1 during pain [36–38]. In addition, Nash and his investigators revealed that the orofacial muscle pain was associated with signal intensity increase in M1 [39]. Consistent with the above findings, the increase in the DC values of the RPG shown in this study may reflect a significant pain-related activation in this region.

A series of studies have shown that the MTG is related to the retrieval of lexical and syntactic information [40–42]. Previous research has shown that the cerebral activity was significantly increased in the middle temporal and superior temporal areas when the tooth is stimulated [25]. In a study that simulated dental treatment, participants' bilateral temporal lobes were significantly activated and corresponding functional activities were significantly enhanced [34]. Similarly, Ettlin and his associates [8] found that strong activation was localized at the inferior and middle temporal gyrus during dental stimulation. Moreover, in a study of acupuncture responses, participants showed significant activation of temporal lobe and increased functional activity in that cerebral regions [43, 44]. In addition, other studies have shown that the functional state of the temporal gyrus is related to emotional control such as anxiety and depression (negative emotions) [45, 46]. In our study, TA patients had significantly increased DC values in the LMTG compared with normal individuals. The linear regression analysis showed that the DC value of the LMTG of TA patients was positively correlated with VAS. Thus, the pain experienced by TA patients was associated with a significant increase in functional activity in the LMTG, a cerebral region that experienced significant stimulation.

The mean DC value of specific ROI was collected for ROC curve analysis, providing an effective differential diagnosis method for the identification of diseases and healthy groups. And this method analyzed sensitivity and specificity as statistical indicators. If the AUC is 0.5-0.7 and 0.7-0.9, respectively, the accuracy is considered low and high. In the cerebral areas involved in this study, ROC curve analysis revealed that all of them had high differential diagnostic significance including RLG, RPG, and LMTG, indicating that DC values could serve as promising biological indicators for distinguishing patients with TA from HCs. Furthermore, from what we have discussed above, here is a summary of the function of the above cerebral regions and the effects of the corresponding dysfunction (Table 4).

However, this study has the limitations of small sample size. In order to obtain more accurate results, we need to expand the sample size in the following studies. Second, the inclusion criteria were not rigorous; both acute toothache and chronic toothache were not further differentiated. In addition, there was no significant classification of odontogenic and nonodontogenic pain in the inclusion criteria for patients with TA. On the other hand, there are a variety of causes for tooth pain, and further research is needed to make a more rigorous distinction between the inclusion criteria. Despite the above defects in our study, the underlying pathogenesis of TA is still revealed to be related to abnormal activity and functional changes in specific cerebral regions.

## 5. Conclusion

To sum up, our neuroimaging study suggests that there are alternations in spontaneous cerebral functional activities in certain cerebral regions of TA patients. Therefore, this study provides a basis for an in-depth understanding of the underlying neuropathologic mechanism of TA patients. Moreover, the change of DC values in specific cerebral regions can be used as one of the effective indicators to detect pain-related diseases.

## Figures and Tables

**Table 1:** DC method applied in pain-related diseases.

Authors	Year	Diseases
Lee et al. ^[^15^]^	2019	Chronic migraine
Liu et al. ^[^16^]^	2019	Persistent somatoform pain disorder
Wang et al. ^[^17^]^	2017	Acute unilateral open globe injury
Yan et al. ^[^18^]^	2020	Chronic shoulder pain
Zhang et al. ^[^19^]^	2019	Primary open-angle glaucoma

Abbreviation: DC: degree centrality.

**Table 2:** Demographics and behavioral results of TA and HC groups.

	TA	HC	*t*-value	*p* value
Male/female	8/10	8/10	N/A	>0.99
Age (years)	41.18 ± 11.65	40.73 ± 12.48	0.088	0.679
Handedness	18 R	18 R	N/A	>0.99
Duration (months)	2.04 ± 1.08	N/A	N/A	N/A
VAS	6.36 ± 1.49	N/A	N/A	N/A

Independent *t*-tests comparing the two groups (*p* < 0.05 represented statistically significant differences). The data are shown as the mean ± standard deviation. Abbreviations: TA: toothache; HC: healthy control; N/A: not applicable; R: right; VAS: visual analogue scale.

**Table 3:** Brain regions with significant differences in DC between TA patients and HCs.

L/R	Brain regions	BA	MNI coordinates			Peak voxels	*t*-value
			*X*	*Y*	*Z*		
R	Lingual gyrus	18	18	-72	-3	31	6.1635
R	Precentral gyrus	3.40	30	-39	54	20	6.5673
R	Precentral gyrus	3.2	21	-33	81	52	6.6338
L	Middle temporal gyrus	39.19	-42	-69	12	49	6.8246
L	Middle temporal gyrus	39	-51	-57	6	20	6.0745

The statistical threshold was set at voxel with *p* < 0.01 for multiple comparisons using false discovery rate. Abbreviations: DC: degree centrality; BA: Brodmann area; HC: healthy control; MNI: Montreal Neurological Institute; R: right; L: left; B: bilateral.

**Table 4:** Brain region alternation and its potential impact.

Brain regions	Experimental result	Brain function	Anticipated results
Right lingual gyrus	TA>HCs	Logical analysis, visual memory encoding, and emotional control	Impact visual processing and attentional system, depression, and anxiety
Right precentral gyrus	TA>HCs	Motor control and associated with the perception of pain	Left somatosensory disorder
Left middle temporal gyrus	TA>HCs	Pain perception and emotional control	Somatosensory disorder, depression, and anxiety

Abbreviations: HCs: healthy controls; TA: toothache.

## Data Availability

The data used to support the findings of this study are available from the corresponding author upon request.
